# Geostatistical spatial projection of geophysical parameters for practical aquifer mapping

**DOI:** 10.1038/s41598-022-08494-5

**Published:** 2022-04-06

**Authors:** Jagriti Dabas, Sarah Sarah, N. C. Mondal, Shakeel Ahmed

**Affiliations:** 1 TERI-SAS, Vasant Kunj, New Delhi, India; 2 Aquifer Mapping Group, CSIR-National Geophysical Research Institute, Hyderabad, 500007 India; 3 Department of Earth Sciences, University of Kashmir, Srinagar, 190006 India; 4 Earth Process Modeling Group, CSIR-National Geophysical Research Institute, Hyderabad, 500007 India; 5 Academy of Scientific and Innovative Research (AcSIR), Ghaziabad, 201002 India; 6 Maulana Azad National Urdu University, Hyderabad, 500032 India

**Keywords:** Hydrology, Statistics, Geophysics

## Abstract

Dense data acquisition for 3-D high-resolution aquifer mapping through heliborne transient electromagnetic (HTEM) survey is continually not possible due to various technical and administrative constraints. Consequently, we apply ground geophysical surveys at possibly closer spacing to collect the sub-surface information in the no-fly area, which provides only a regional aquifer picture. In the area near Patna of Northern India, an extent of 18% was covered under the HTEM survey, and the rest was surveyed by ground geophysical methods. Both data are integrated using the theory of regionalized variables. The parameters of multi-aquifers i.e., top of the first aquifer, top of the separating clay layer, top and the bottom of second aquifer, are estimated together with their respective resistivities. The estimations are made at an interval of 250 m, practically an appropriate distance at which dense data generation was carried out using the HTEM survey. The integrated approach generates the data in the no-fly area with the same spatial density as the flown area. With this, we achieved the goal of completing the 3-D aquifer mapping of the entire area with dense data at high spatial resolution. This is a unique finding to manage the handicapped situation in this HTEM surveys, and an aide to overcome such constraints with cost-effectiveness.

## Introduction

India as a whole is still not a water-scarce country compared to many others in the world but is facing many water-related issues due to the rising water demand, unplanned management, and uneven distribution of its water resources^1^. Water resources management in densely populated areas with stressed aquifer systems in the Ganges basin of Northern India is a huge challenge that requires complete knowledge of the aquifer system^2,3^. High-resolution aquifer mapping is important in such aquifer systems to understand their hydrogeological functioning and achieve sustainable groundwater management. In India, aquifers exist in the major geological formations, which are extremely variable and complex with limited information making it difficult to assess and manage them^4^. Several ground geophysical methods like seismic, magnetic, electrical, and electromagnetic are commonly practiced, but among them, Vertical Electrical Soundings (VES), Electrical Resistivity Tomography (ERT), and Transient Electromagnetic (TEM) surveys have been extensively used for groundwater studies, especially in deducing aquifer geometry, resistivity/electrical conductivity of the subsurface, mapping of the intrusive bodies, groundwater quality, etc^5^. Among all geophysical methods, electrical and electromagnetic methods that provide a measure of formation resistivity or conductivity, are the most commonly applied methods for groundwater exploration^6,7^. The ground-based electrical and electromagnetic methods, though very effective for formation characterization with large-scale applications, quite often are limited by various field constraints viz., inaccessible terrain, non-availability of suitable space, and are also time-consuming. Thus the non-invasive heliborne transient electromagnetic (HTEM) investigations with the ability to acquire a large amount of closely spaced data at a fast pace and relatively low cost, have been gaining global popularity for hydrogeological studies during the past few decades^8–10^. In the recent past, this HTEM with a wide range of applications has been established as a potential mapping tool to provide the spatial distribution of discontinuous permafrost^11^, aquifer structures^12–14^, seawater intrusion in coastal aquifer^15^, aquifer below the ice cover^16^, a network of bedrock fractures in hard rock area^17^, and paleochannels in parts of the world^8,18^.

**Figure 1:**
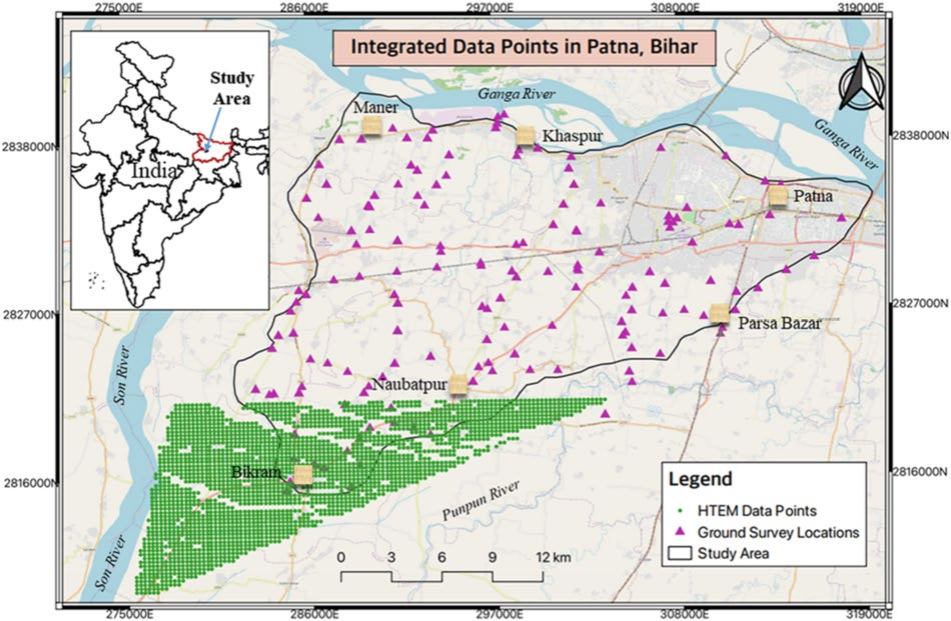
Study area showing the survey points of ground geophysics and HTEM in a part of Ganga basin, Patna, Northern India (N.C.M.: sketched this figure with the help of ArcGIS Desktop 10.7, http://www.esri.com).

As in the case of any geophysical technique, HTEM survey also has several constraints, mainly that the instrument used during flight is suspended in air, hence flying is not safe over any settlement area, particularly in urban areas with high-rise buildings leading to data gaps in the survey. Due to some technical and administrative constraints, thorough flying could not be conducted in the entire area of interest. As a result, in our case, only 18% of the study area was covered by the HTEM survey in Northern India (Patna) under the pilot aquifer mapping (Fig. 1). Therefore, data density was quite sparse, and modeling the aquifer system in the entire area of 521 km^2^ on the same spatial grid became a strenuous task. However, several ground surveys were then deployed, which could not match the data density as in the case of the HTEM survey as the spatial frequency of data acquisition in the case of the HTEM survey is always higher as compared to the ground geophysical surveys.

Due to the curtailment of cost and feasibility, parameters cannot always be measured at a large number of locations as may be required by an aquifer model. Therefore, it is ineluctable that various parameters must be estimated at the unmeasured locations from the computed and interpreted values using a mathematical tool^19^. Geostatistics, based on the theory of regionalized variables^20^, has found applications in almost all the domains of hydrogeology from parameter estimation to extrapolative modeling for groundwater management, e.g., parameters estimation at unmeasured locations, groundwater model construction (optimal discretization), unbiased model calibration using estimation errors and in acknowledging the best models for prediction^21^. Data gaps can be completed with geostatistical estimation with the help of unbiased kriging estimation variance minimization technique known as Best Linear Unbiased Estimator (BLUE)^22^. The advantage of kriging is that in the absence of estimation error, it is an exact interpolator at measured points^23^. In other words, Kriging is an estimation technique of making optimal, unbiased estimates of regionalized variables at unmeasured locations using the variogram analysis and the input data values^24^. The Kriging takes into account the spatial formation of the parameter, and it hence has the edge over other methods like arithmetic mean method, nearest neighbor method, distance weighted method, and polynomial interpolation. Also, estimated variance is computed by kriging at every estimated point, which is an indicator of the accuracy of the predicted values. This is considered as the significant advantage of kriging over other estimation techniques^25^.

This study addresses the spatial variability of aquifer systems in the Ganga basin covering Patna region of Bihar (Northern India), as shown in Fig. 1. In the study area, the majority of the portion is covered by the alluvial-aquifer system in Middle Ganges Plain where we encounter the two to three-layered aquifer systems separated by thick confining clay layer^26–28^. Kriging as a geostatistical estimation technique has been relevantly applied in a spatial projection of a target variable, i.e., depth of the geological formation layers in the aquifer system with their corresponding resistivity values in the entire study area that includes both the area from HTEM and ground surveys using their variability from the limited measurements at unmeasured locations. A range of estimated values is provided using the variance of the estimation errors for the above-targeted variables. The predicted results generated data in the no-fly area with the same spatial density as of HTEM flown area. Further 3D modeling of the aquifer geometry was carried with their respective resistivity values overlaid as contours. This has helped in completing the aquifer mapping of the 427 km^2^, the non-flown area with dense data at a high spatial resolution.

## Results and discussion

### Variographic analysis for depth of the aquifer

**Figure 2:**
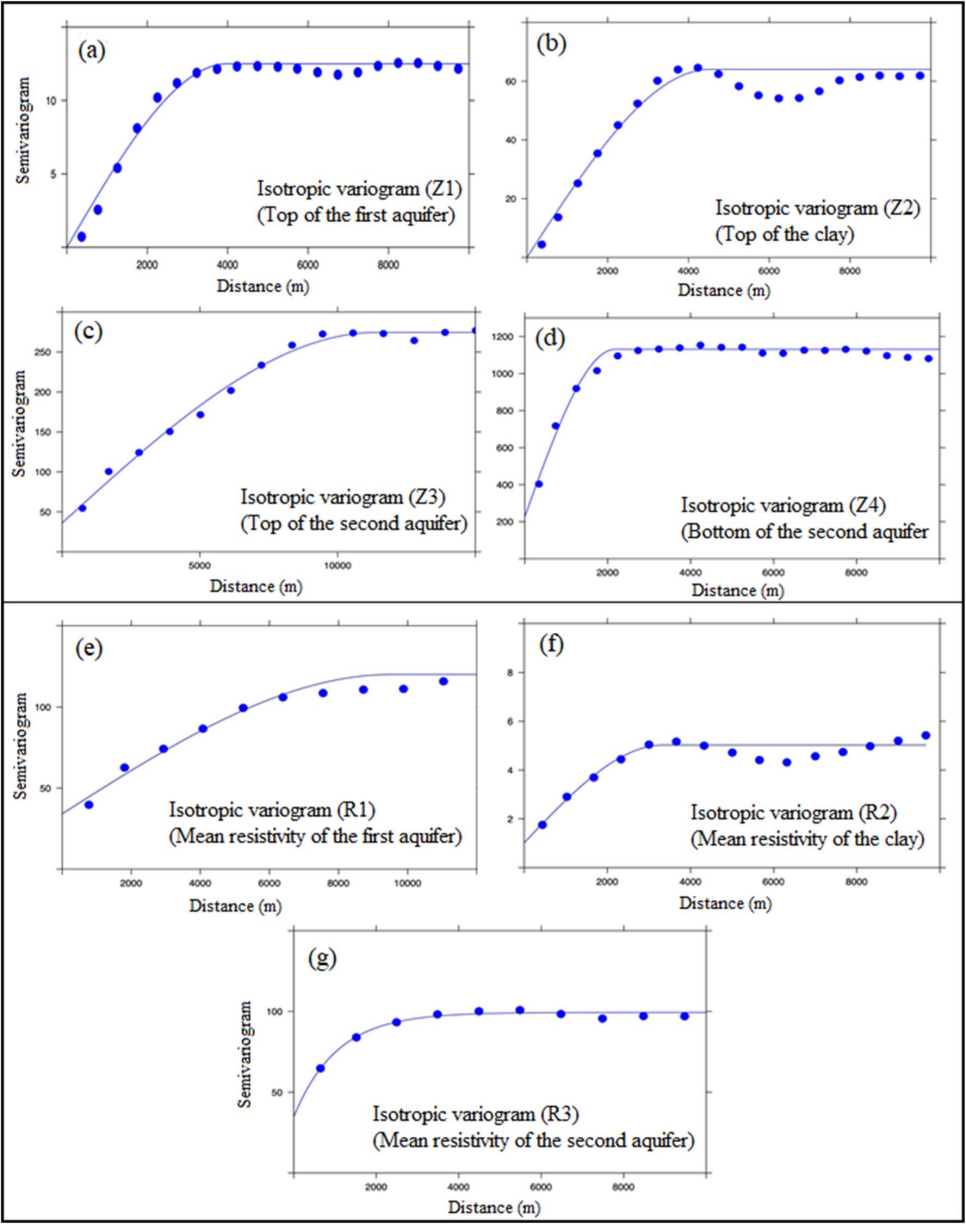
Variograms of (**a**) Z1: top of the first aquifer, (**b**) Z2: top of clay, (**c**) Z3: top of the second aquifer, (**d**) Z4: bottom of the second aquifer, (**e**) R1: mean resistivity of the first aquifer, (**f**) R2: mean resistivity of the clay, and (**g**) R3: mean resistivity of the second aquifer of multi-aquifer system in a part of Ganga basin, Patna, Northern India.

After the quality check, experimental variograms were plotted for all four depth parameters. The theoretical model fitting was executed with the help of “R” statistical software. Four variograms were plotted for each depth parameter, as shown in Fig. 2a–d. By visible interpretation and considering RMSE (close to 1.0) and R^2^ (> 0.7) values (as per the Eqs. 1–4), spherical models were chosen as the best-fitted models as compared to other models as it resulted in minimum standard error. A good data density made possible to estimate the small-scale variability in the variograms, and no nugget effect was found in any of the variograms, implying the least measurement error^29^.

The cross-validation results (presented in Table 1) calculated were in accordance with ideal statistics (Eqs. 1–4), hence proved to be significant. As seen in Table 2, Z1 corresponds to minimum root mean square error (RMSE) of 1.73, R^2^ = 0.92, and mean error (ME), mean square normalized error (MSNE) and mean squared prediction error (MSPE) are also appropriate. The prime reason for such implications is the data input for Z1, which was highest in number as compared to the other 3 parameters, and also variance in the data set was least. Also, Z4 i.e., the base of the second aquifer, exhibits the most variance (Table 1), maximum RMSE of 24.25, and minimum R^2^ value of 0.70 (Table 2), giving rise to the conclusion that data input and quality for Z4 was least amongst all. The above cross-validation results show that the chosen model and its parameters are adequate. The model fitting for the empirical variogram was carried out using the weighted least square method, and the search radius for three parameters was 10 km, but for Z3, it was 15 km because of the higher data density in the HTEM surveyed area.

**Table 1:** Descriptive statistics of the cross-validation for depth of the aquifer corresponding resistivity.

Layer depth	Minimum	Mean	Median	Maximum	Variance	CV
**Layer depth (in m)**						
Z1	4.90	35.34	35.24	52.20	20.68	0.12
Z2	− 39.7	− 5.5	− 4.1	19.9	89.16	− 1.71
Z3	− 108.9	− 53.87	− 54.6	− 12	210.004	− 0.26
Z4	− 252.1	− 179.2	− 178.4	− 61.6	1227.38	− 0.195
**Layer resistivity (in Ohm m)**						
R1	10.4	44.6	45.3	120	160.81	0.28
R2	8.2	13.51	13.1	19.9	5.80	0.17
R3	18.20	46.16	45.60	105.10	124.40	0.24

**Table 2:** Descriptive statistics for the depth values and the cross-validation for resistivities of the aquifer layers.

Layer depth	RMSE	R^2^	ME	MSPE	MSNE
**For layer depth (in m)**					
Z1	1.73	0.92	− 0.02	3.01	3.27
Z2	1.96	0.97	− 0.009	3.86	0.93
Z3	5.76	0.91	0.03	33.20	0.70
Z4	24.25	0.70	0.23	588.23	1.38
**For layer resistivity (in Ohm m)**					
R1	6.45	0.86	− 0.04	41.60	1.36
R2	1.35	0.82	0.01	1.84	1.11
R3	7.45	0.74	0.04	55.51	1.01

The variogram interpretations lead us to an observation in the structure of the variogram pattern, which showcases the occurrence of one of the natural phenomenon in the study area. The periodic pattern was seen in all the variograms (Fig. 2a–d), which implies that paleo-channel and “Son river” flowing across that area possibly occur there. This can be further confirmed by the spatial autocorrelation showing the distance from where this paleo-channel is occurring and investigations comparing the original geomorphological map (Fig. S1).

### Variogram analysis for resistivity of the aquifer

The variogram analysis for resistivity values inferred that there exists some nugget effect in the data (Fig. 2e–g), which is due to the fact that subsurface geological formations may not be continuous and can change non-uniformly. Therefore, abrupt change in resistivity values from point to point resulted in the nugget effect. The possible aquifer contamination can also change the resistivity values drastically, hence the nugget effect is seen. The variance or sill (Table 1) seen in the variograms is least (5.80) for the resistivity of clay because the resistivity of clay is less than 15 Ω-m and hence gives less variance in the data. The cross-validation statistics calculated (Table 2) are satisfactory and in accordance with Eqs. 1–4, but not as compared to the depth values because of the pertaining nugget effect. The same periodic pattern is visible in these variograms as well, justifying the fact that there exists a hydrogeological phenomenon in the form of paleo-channel flowing across that area.

### Predictions for the depth of the aquifer using ordinary kriging

After the model fitting for the variogram, ordinary kriging was applied as a geostatistical estimation procedure using Eqs. (9) to (12). The spatial grid used for generating the prediction maps was made by taking 250 m grid resolution. The same projection system was applied to the boundary and spatial data to avoid error in projecting the data. The geostatistical estimation as mentioned in the aforesaid section was implemented using package “rgdal”^30^ and “sp”^31,32^ using an open-source statistical software “R”^33^ and R Studio, version 0.99.893^34^.

**Figure 3:**
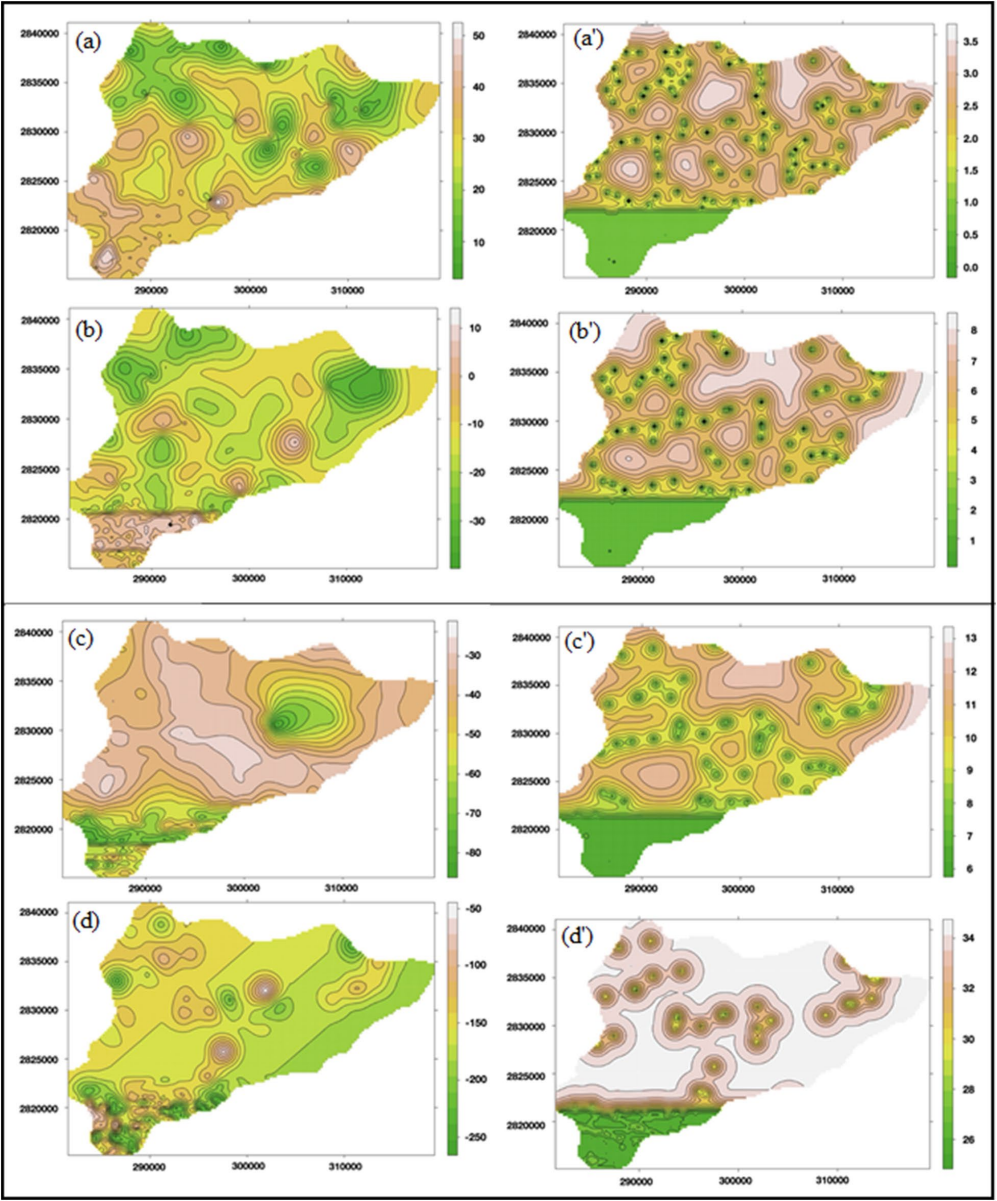
Predicated Z-values of (**a**) Z1, (**b**) Z2, (**c**) Z3, and (**d**) Z4 (in AMSL) corresponding their errors in the cases of (**a′**) Z1, (**b′**) Z2, (**c′**) Z3, and (**d′**) Z4 (in AMSL), respectively (J.D.: drawn this figure with the help of R Core Team (2013). R: A language and environment for statistical computing. R Foundation for Statistical Computing, Vienna, Austria, http://www.R-project.org/).

Prediction maps, as shown in Fig. 3a–d were generated using terrain colors (color theme in R statistical software). All the values of aquifer depths were taken above mean sea level (AMSL), also known as reduced level of the aquifer since the topography effect was to be taken into consideration. The maps obtained were completely in accordance with the terrain of the study area. Although data were sparsely distributed in the non-flown area however with the technique of regionalized variables, good predictions were achieved throughout the study area with a spatial resolution of 250 m. The error maps (Fig. 3a′–d′) followed by prediction maps guided us about the variance of estimation error or broadly known as sigma (**σ**). One of the most important advantages of kriging is that it provides the amount of estimation error from which statistically significant confidence intervals are stated.

As seen in the prediction maps and descriptive analysis table (Table 2), it is easily to observe and compare the depth values obtained. The depth of the first aquifer provided the best results with the least sigma values ranging from 0 to 3 m (σ), which implies 95% confidence interval for the estimations made. With the increasing depth, estimation error increased slightly because the variance increases with depth (Table 2). The data on aquifer depth information for layers other than the first aquifer was comparatively less in number as ground-based surveys could not resolve the merging of 2 aquifers, only HTEM data provided information about the second aquifer^26–28^. A lot of variation is observed, since it lies deeper, the exact depth information could not be extracted due to the two merging aquifers below the ground. The HTEM data provided the aquifer information till 300 m depth below ground level (bgl), whereas ground surveys like GTEM, VES, ERT, and e-log data provided the information till the clay layer on most of the points, as shown in Figs. S2–S5. Further, data was taken till the depth of investigation (DOI) manually because of the merging of aquifers. Figure 3a depicts the pattern of depth values in south-western and central regions similar to the geomorphological map, as shown in Fig. S1. This relates to the existence of paleo-channels in the study area which are confined within 20–30 m depth from the ground surface and 40–50 m above mean sea level^26–28^. The aforesaid facts prove the similarity between variogram analysis, prediction maps, and geomorphological map, thus justifying the kriging results as appropriate.

### Predictions for the aquifer resistivity using ordinary kriging

**Figure 4:**
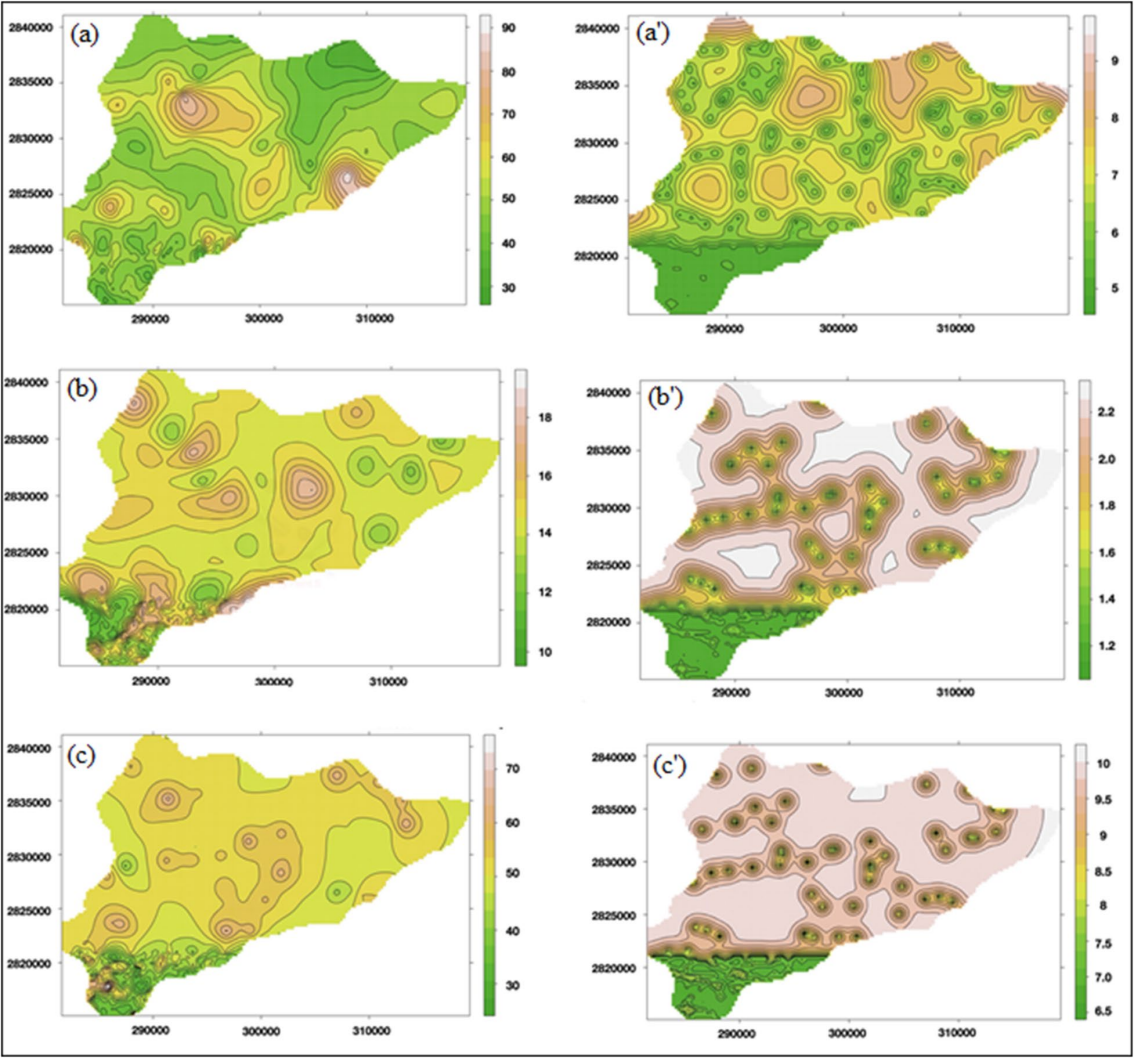
Predicated R-values of (**a**) shallow aquifer (R1), (**b**) intermediate clay layer (R2), and (**c**) deeper aquifer (R3) in Ohm-m corresponding of the predicted R-values as (**a′**) R1, (**b′**) R2, and (**c′**) R3 (in Ohm-m), respectively (J.D.: sketched this figure with the help of R Core Team (2013). R: A language and environment for statistical computing. R Foundation for Statistical Computing, Vienna, Austria, http://www.R-project.org/).

The quality of the aquifer water is represented by the resistivity values below the earth’s surface. Therefore, to depict a complete aquifer system including quality and quantity, respective resistivity prediction maps (Fig. 4a–c) were generated. The sigma values obtained are quite significant, as seen from the maps (Fig. 4a′–c′), which range between (0–9 Ohm m). The descriptive statistics (Table 1) for the predicted resistivity values shows clearly the range of data in the entire area, earlier restricted to a limited area. The resistivity values for clay showed minimum sigma because of the range and data values of resistivity, ranging between 0 and 15 Ohm-m. The least variance of resistivity of clay (~ 5.80 Ohm-m) amongst all 3 resistivity values resulted in minimum estimation error or standard deviation of kriging estimation. Resistivity values for the first aquifer and second aquifer are almost the same (90 Ohm-m) because of the water storing capacity. The higher resistivity of the aquifer indicates higher granularity and higher water yielding capacity (as shown in Figs. S2b,c–S5)^26,27,35^.

Again, the similar pattern represented by resistivity values in Fig. 4a corresponds to the occurrence of paleo-channels in the western region. The paleo-channels comprise of material with relatively higher granularity associated with higher resistivity values compared to the surroundings where the granular material of paleo-channels is saturated with fresh water. To maintain the similarity, terrain colors have been used, and all the map generation have been done in R statistical software.

### 3-D modeling of the aquifer system

This section consists of three parts.

i. Estimation of the true variability of the parameters using the variable density data that are required to obtain the variograms with an adequate number of pairs.
ii. The estimated values at the unmeasured points at reasonably high density in the non-flown area to match the data acquisition with the HTEM survey in the flown areas. The results are in the form of estimated values as well as their variance of the estimation errors (VEE). This error provides the range within which the true values lie. Hence provides the freedom to adjust the values within the range required to achieve the desired result.
iii. An additional exercise was carried out to demonstrate the advantages of employing geostatistical estimation. The map of all four layers is compared after the geostatistical estimation with the contour of the corresponding map without applying the geostatistical estimation. The comparison clearly demonstrates the advantage.

The first and most part has been the estimation of the true variability of the seven parameters considered, and the data availability of different densities, provided an accurate variogram and true variability. All the 4 layers were contoured and put together in a 3D view to provide a 3D aquifer geometry in the entire study area as an initial objective of the study.

**Figure 5:**
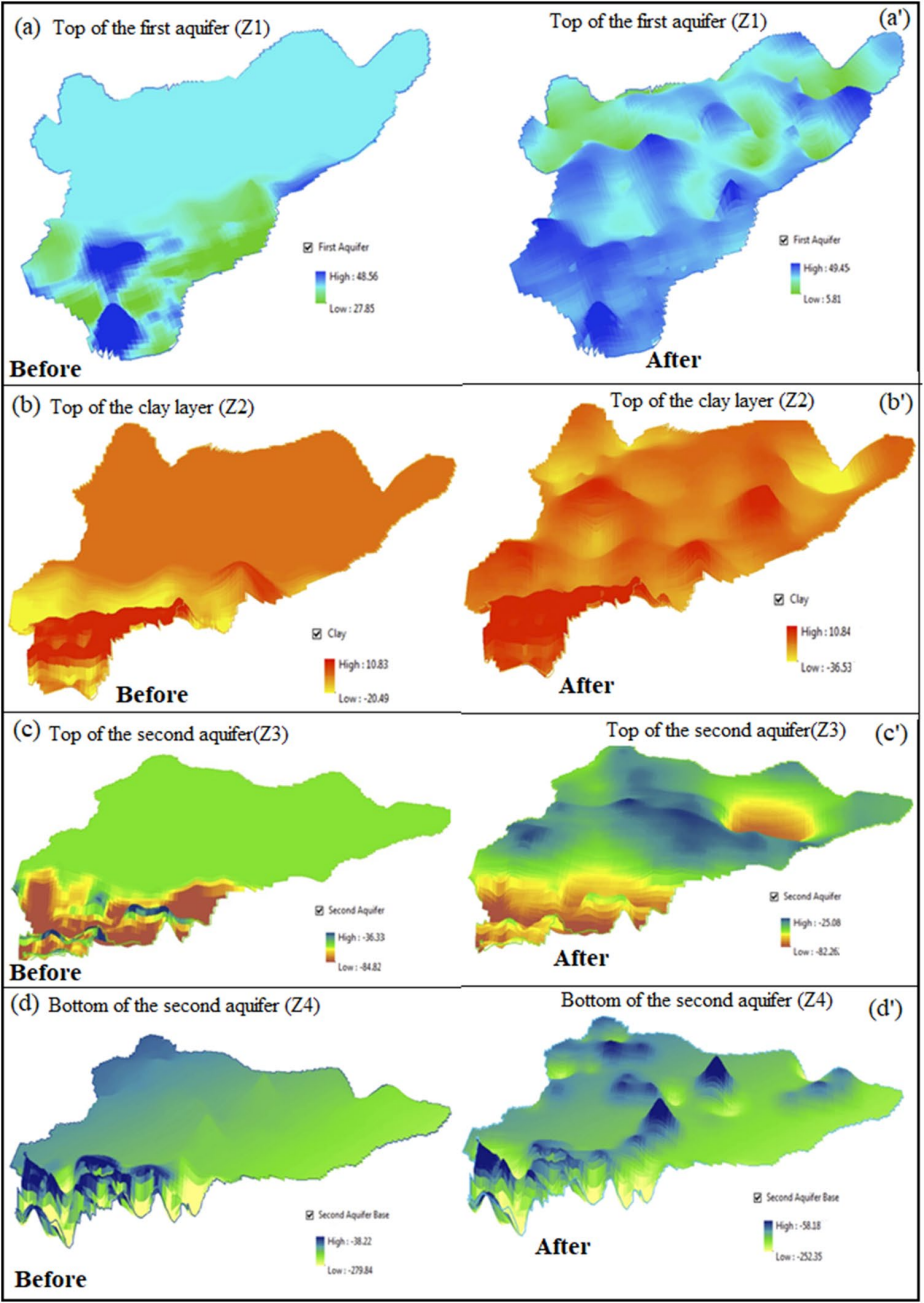
Comparison of 3D aquifer geometry for (**a**–**a′**) top of the first aquifer (Z1), (**b**–**b′**) top of clay layer (Z2), (**c**–**c′**) top of the second aquifer (Z3), and (**d**–**d′**) bottom of the second aquifer (Z4) (J.D: drawn this figure with the help of ArcGIS Software, Version 10.0. Redlands, CA: Environmental Systems Research Institute, Inc., 2010, http://www.esri.com).

The depiction of 3-dimensional models of the depth and resistivity values of the aquifer was one of the most important tasks, and the crux of the study could easily be seen from Fig. 5. Before and after images imply that after execution of the technique, data was successfully generated in the non-flown area with their respective range values.

Each figure depicts the overall change in the study area. Therefore, it implies that after the geostatistical estimation of the geophysical parameters, the main objective i.e., data generation with the spatial frequency of 250 m in the entire area was successful. Although dense data with high spatial resolution were available only in the 18% of the area, data conditioning using geostatitics provides a better mapping of regional geological aquifer structure in the area with data gaps.

**Figure 6:**
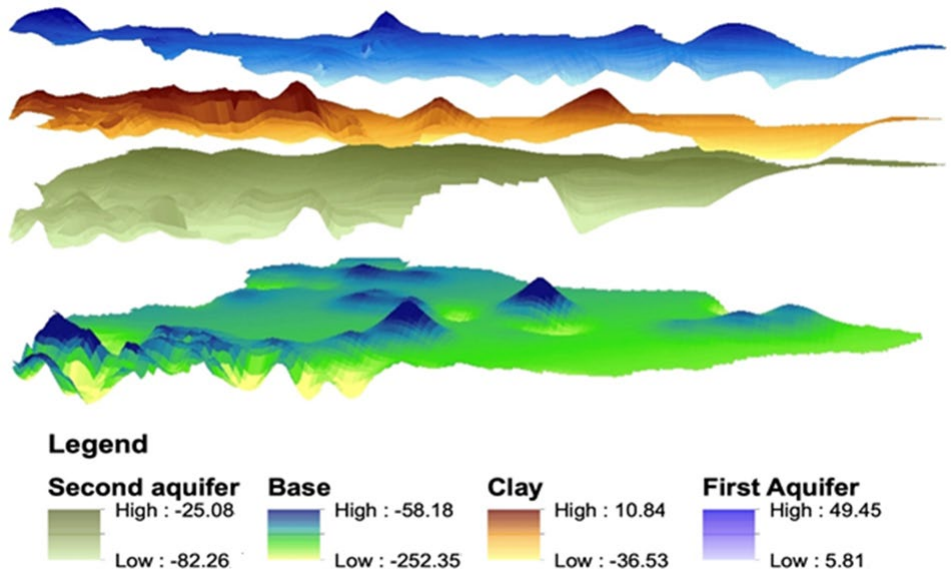
Combined 3D aquifer system of the study area (J.D: sketched this figure with the help of ArcGIS Software, Version 10.0. Redlands, CA: Environmental Systems Research Institute, Inc., 2010, http://www.esri.com).

Further digital depth model (DDM) depicted the geometry of the aquifer system, as shown in Fig. 6, and could be seen as a whole for the entire study area. This DDM interprets the thickness of the aquifers and the confining layer (clay), and depicts that the depth values of each succeeding layer are not overlapping at any particular point. The 3D model depiction provided an added rationale for the kriging estimations because of the aforesaid fact of a non-overlapping model. If the estimated depth values would have been in a larger error window, then overlapping of subsequent layers was certain. Further, aggregating all the results and estimations of the kriging technique as discussed in earlier sections about cross-validation statistics, estimated depth values and corresponding sigma, similarity with geomorphological map, and variogram analysis, it can be concluded that the data generation to fill data gaps with the help of geostastistics was executed successfully with minimal errors.

## Conclusions

It is quite common that permission for flying over some restricted areas for aquifer mapping is not granted due to several administrative, technical, and security reasons. However, the parameters of the natural system are required everywhere in the entire study area to capture the true variability. Thus, the ground survey is carried out to fill these gaps, but the density of data collected through ground geophysical methods is not comparable to the high resolution data provided by the HTEM surveys. To overcome this, the geostatistical technique was applied to estimate the parameters on the unmeasured points and match the data density with a high-resolution HTEM survey. The layered parameters of the aquifer system viz., thickness, and the resistivity of the different layers, were estimated using the theory of regionalized variables. The most important part of this exercise was to determine the precise variability of these parameters due to the given special arrangement of measurement points, providing numerous numbers of pairs at a shorter distance lag, etc.

The nature of the data available from different sources was for the same variable but at different spatial intervals. This has been an advantage for calculating the variograms as the pairs were available at very short intervals as well as long intervals. Thus, a well representative variogram was calculated as evident from Fig. 2, and easily modeled to obtain the theoretical variograms. Despite very smooth variograms reasonably representing the true variability of all parameters, the cross-validation tests were carried out to double-check the parameters of the theoretical variogram (Table 1). The estimation of variogram represented their true variability due to the presence of unprecedented high-density data in the area flown. In addition, data coming from various ground surveys added the best possible network for estimating a variogram; a large number of pairs with smaller lags then sufficiently good numbers for intermediate as well as larger lags. This is the reason why the cross-validation test has become simple and satisfactory results were obtained after a very few iterations. Also, the inspection of the periodic pattern of variograms indicated the presence of paleo-channel naturally occurring in the study area, which was also reflected in the final prediction map (Fig. 5) and a depth pattern that was confined within 20–30 m from the ground surface. This indication could be validated by the comparison with the geomorphological map (Fig. S1). Further, additional information about the distances from where the paleo-channel existed could be derived from spatial autocorrelation. The digital depth model (shown in Fig. 6) also exhibited that there was minimal overlap between the estimated aquifer layers, thus supporting the aforesaid facts and making the study more concrete and practical. If at all, the layers in Fig. 6 overlap or do not fit in the conceptual as well as geological model, one may take advantage of the error maps (for example, Fig. 3a′–c′) to adjust the layers. Since the true value of the parameter lies in the interval range of − 2σ to + 2σ known as the standard deviation of the estimation error, which has a 95% confidence level, therefore error maps were helpful in the modification of overlapped layers.

However, the results represent only a first step towards the extraction of sub-surface information for the aquifer units. The technique developed in this study not only provides the best-estimated value to fill the data gap but can also be applied to save the time and cost involved in the HTEM survey for the data acquisition. The problem of HTEM survey that cannot be conducted around densely populated areas can be easily resolved using this methodology. In consequence, this technique can provide resonance to the data acquisition process producing high-density data sets, even in highly populated areas, where the HTEM survey is not feasible.

## Methodology and data

### Geostatistics for estimation of layer parameters

Enstatites, the theory of the regionalized variables initially was developed for mining and the ore reserve estimation, but soon it has found applications in many fields of earth and natural sciences as any natural variable can be categories as regionalized variable. It has rigorously been applied in hydrogeology or groundwater hydrology as evident from the work of Delhomme^23^. The use of geostatistics in the spatial analysis of natural variables has become increasingly well-known with more and more applications. According to Yamamoto^36^, Ordinary Kriging has been commonly applied as a dependable geostatistical method. Kriging is discerned from Inverse distance weighting (IDW) and many other interpolation methods by considering the variance of estimated error of the parameters^37^. Several applications of ordinary kriging had been executed for environmental studies^38,39^. Ahmed and Murali, and Roth et al. are amongst several others who have made extensive use of geostatistics in groundwater hydrology^40,41^.

The steps contained in Kriging applied to hydrogeological parameters are: the transformation of original variables if required, variographic analysis (i.e., structure analysis), cross-validation tests, and estimation. The accuracy of the estimation depends on variographic analyses that consist of capturing the spatial variability of a parameter. The variogram should be carefully be finalized as it controls the way the kriging weights are assigned to data values during the estimation and hence controls the quality of the results. The procedure involves the calculation of experimental Variogram; it’s modeling by fitting allowed theoretical variogram functions like linear, Gaussian, exponential and spherical, etc., and its validation by estimation on points where the available original/field data. Cross-validation tests are performed as variograms obtained by modeling an experimental variogram are often quite approximate. Cross-validation is performed by predicting the random function at the points where realizations are already available (i.e., at data points) and making ensemble comparisons with the estimated data^42^. Measured values are removed from the data set one by one, and the same is repeated for the entire data set. Thus, at all the measurement points, the measured value (z), the estimated value (z*), and the variance of the estimation error (σ^2^) are available. Following statistics are calculated on an average basis and the variogram that satisfies them is finalized.

1 $$ \frac{1}{N}\sum\limits_{i = 1}^{N} {\left( {z_{i}^{obs} - z_{i}^{*} } \right) \approx 0.0} $$

2 $$ \frac{1}{N}\sum\limits_{i = 1}^{N} {\left( {z_{i}^{obs} - z_{i}^{*} } \right)^{2} \approx \min } $$

3 $$ \frac{1}{N}\sum\limits_{i = 1}^{N} {\left( {z_{i}^{obs} - z_{i}^{*} } \right)^{2} /\upsigma _{i}^{2} = 1} $$

4 $$ \left| {z_{i}^{obs} - z_{i}^{*} } \right|/\upsigma _{i} \le 2.0\quad \forall i $$

Various parameters of the variogram model are gradually modified to obtain satisfactory values of Eqs. 1 to 4. Ideal cross-validation results and their interpretation as per the mentioned equations are as follows:

i. Equation 1 corresponds to mean error (ME), which should be ideally zero. It depicts the mean of residuals (difference between observed and predicted values). If the estimated results are unbiased then the ME will be approximately Zero^43^.
ii. Equation 2 refers to Mean Square Prediction Error (MSPE). It denotes the mean of the squared residuals that depicts the prediction error. This value should ideally be small.
iii. MSNE refers to the Mean Squared Normalized Error, which is represented by Eq. (3). It denotes the ratio of the errors from two different sources so should be ideally close to 1.
iv. Another statistic that is helpful to assess the quality of prediction is Root Mean Squared Error (RMSE). It measures the difference between the predicted and the observed values, and these individual differences are also called residuals^44^.

5 $$ {\text{RMSE}} = \surd \frac{1}{{\mathbf{N}}}\mathop \sum \limits_{{{\mathbf{i}} = 1}}^{{\mathbf{N}}} \left( {{\mathbf{z}}_{{\mathbf{i}}}^{{{\mathbf{obs}}}} {-}{\mathbf{z}}_{{\mathbf{i}}}^{*} } \right)^{2} $$

v. And R^2^ denotes the correlation between the predicted and observed values. Its value ranges from − 1.0 to + 1.0, and a value above 0.6 is considered to be statistically significant.

### Spatial prediction of aquifer parameters

This specialized study is divided into four steps as follows:

i. Preparation of processed data on layer thickness and the resistivity in the form of input variables to understand the data availability and determine the first-hand variability.
ii. Careful variographic analyses and cross-validation to finalize the variability structure of the given parameter. Modeling of the variograms with authorized theoretical models.

A generalized formula to calculate the experimental variogram from a set of scattered data can be written as follows^19^.

6 $$ \gamma (\underline{d} , \underline{\theta } ) = \frac{1}{{2N_{d} }}\sum\limits_{i = 1}^{{N_{d} }} {} \left[ {z\left( {x_{i} + \hat{d},\hat{\theta }} \right) - z\left( {x_{i} , \hat{\theta }} \right)} \right]^{2} $$

7 $$ {\text{where}},\;d - \Delta d \le \hat{d} \le d + \Delta d,\theta - \Delta \theta \le \hat{\theta } \le \theta + \Delta \theta $$

8 $$ {\text{with}}\;\underline{d} = \frac{1}{{N_{d} }} \sum\limits_{i = 1}^{{N_{d} }} {\hat{d}_{i} } ,\;\underline{\theta } = \frac{1}{{N_{d} }} \sum\limits_{i = 1}^{{N_{d} }} {\hat{\theta }_{i} } $$

where d and θ are the initially decided lag and direction of the variogram with Δd and Δθ as tolerance on lag and direction, respectively. The d and θ are final lag and direction for the calculated variogram respectively. N_d_ is the number of pairs for the lag d and direction θ. The additional Eq. (8) avoids the rounding-off error of pre-decided lags and directions. All the terms are to be carefully accounted for. Δd and Δθ will be zero and d and θ become d and θ, respectively if the data are available on a regular grid, often is the case in mining. The geohydrological parameters also have anisotropy and variograms in that case should be computed by taking pairs in different directions, at least in 2 to 4 directions to ensure the existence or absence of the anisotropy. This exercise becomes feasible when data are available at a large number of points.

A number of approximations are taken while calculating the experimental variogram and this is why the curve of the experimental variogram is not smooth, of course depending on the size of the samples. In any case, a smooth curve with a fixed mathematical function only is allowed to fit through the experimental variogram that is called theoretical variogram. This is to avoid the mathematical problem in solving the kriging estimation equations. The theoretical variogram is defined by two basic parameters called sill and range. In addition, often the small-scale variability is not determined due to lack of close measurements and the theoretical variogram appears to cut the variogram axis with an intercept rather than passing through origin. Although the variogram has to be zero if the distance lag is zero but the apparent intercept is called the nugget effect. In addition, the increase of the variogram curve before becoming flat at the range could be different that provides a few different model types. Thus, in total there are 4 parameters to define a theoretical variogram.

iii. The spatial prediction of depth-wise aquifer information in the entire study area using interpreted data both from the ground and HTEM surveys to generate the high-density values in the entire area where flying was not possible using ordinary kriging.

The value of any variable Z can be estimated at the point x_0_, which has not been measured, the kriging estimate is defined as:

9 $$ Z^{*} (x_{o} ) = \sum\limits_{i = 1}^{n} {\uplambda _{{\text{i}}} {\text{Z}}\left( {{\text{x}}_{{\text{i}}} } \right)} $$

where z^*^(x_0_) is the estimated value at the point x_0_ and λ_i_ are the weighting coefficients to extract the estimated value from the measured values. The λ_I_ are obtained by solving the following equations called Kriging equations^45^:

10 $$ \sum\limits_{j = 1}^{n} {\lambda_{j} }\upgamma \left( {{\text{x}}_{{\text{i}}} ,{\text{ x}}_{{\text{j}}} } \right) +\upmu =\upgamma \left( {{\text{x}}_{{\text{i}}} ,{\text{x}}_{{\text{o}}} } \right) $$

$$ {\text{i}} = 1 \ldots {\text{n}} $$

11 $$ \sum\limits_{j = 1}^{n} {\lambda_{j} } = 1 $$

and the variance of the estimation error becomes:

12 $$\upsigma _{k}^{2} \left( {{\text{x}}_{0} } \right) = \sum\limits_{i = 1}^{n} {\lambda_{i} }\upgamma \left( {{\text{x}}_{{\text{i}}} ,{\text{x}}_{{\text{o}}} } \right) +\upmu $$

In the above set of equations, γ are obtained from the variographic analysis after finalizing the theoretical variogram. After subtitling λ_i_ values to Eq. 9, estimated values are obtained together with the corresponding variance of estimation error from Eq. 12.

iv. To plot 3-D maps using the estimated values as well as the corresponding variance of the estimation errors and obtain the complete picture of the aquifer system. Depth and resistivity were chosen as the target variables in this study to estimate the aquifer geometry in the entire study region.
v. Implication of results to be used in aquifer mapping.

The following seven parameters were used as input variables.

i. Z1—Top of the first aquifer
ii. Z2—Top of clay
iii. Z3—Top of the second aquifer
iv. Z4—Bottom of the second aquifer
v. R1—Mean resistivity of the first aquifer
vi. R2—Mean resistivity of the clay
vii. R3—Mean resistivity of the second aquifer

Depth of the aquifer was chosen as the target variable instead of the middle control point of the aquifer layer and thickness mainly because of two reasons. First of all, we will have to predict only 4 parameters as compared to the former case of 6 parameters, and secondly, the main objective was to estimate the aquifer geometry information without having actually flown in the entire study area and integrating information from all the data source for which most appropriate variable was assumed to be the depth of aquifer system. Resistivity was also chosen as one of the two targeted variables to exhibit respective aquifer characteristics. These two types of variables combined gave an acceptable estimation.

### Geophysical data

Geophysical techniques viz., Vertical Electrical Sounding (VES), Ground Transient Electromagnetic Survey (GTEM), Electrical Resistivity Tomography (ERT), and HTEM surveys together were carried out in the pilot study^26–28^, as shown in Fig. 1. The hydrogeological inputs^35^ were collected and also used in the interpretations of geophysical data to deduce the vertical and lateral extension of the aquifer system to a certain extent. The state-of-art HTEM survey was carried out using the SkyTEM system that utilized low moment (LM: ~ 3000 NIA) and high moment (HM: ~ 490, 000NIA) measurements with a ~ 341 m^2^ transmitter loop that carrying 2 and 12 turns transmitter coils, respectively. The system has the unique feature of applying two separate pluses (dual pulse technology), such as a shorter and longer pulse. The low moment shorter pulse is suitable for shallow investigations, while the longer and more powerful pulse is employed for deeper investigations. These two data sets are synthesized to obtain seamless information on the subsurface from the shallow to the deeper region^46^. A high altitude (> 300 m above ground) test flying was carried out at the beginning and at the end of each flight to measure the primary field response, that is, the effects of the instrument, including the helicopter on the receiver coil. The high-altitude primary field response was used to decouple the system response from the early time gates of low movement data. Thus, the low and high moment measurements added to the system response decoupling from early gates facilities usable HTEM data starting from 4 to ~ 10 µs that results in obtaining high-resolution subsurface characteristics from very shallow to more than 300 m depth.

During the HTEM data acquisition, the helicopter flew with an average flight speed of 22 m/s and an average flight altitude (frame height) of 30 m above the ground, maintaining the flight line spacing of 250 m. There were 50 fly-lines in the west–east direction (Fig. 1). About 770-line km of data was acquired and also accepted for further processing. The data was analyzed using the Aarhus Workbench program for rapid quality check within a couple of hours after its collection. Numerous steps for the detailed processing are presented by various researchers^46,47^. The processing steps include altitude correction, geometrical correction for positioning the measurement points to the center of the transmitter loop, pitch, and roll, primary field correction for the initial data, removal of coupling, laterally constrained inversion (LCI) along the flight line, filtering out the nosy data with high data residual, and finally running spatially constrained inversion (SCI) with a smooth model discretization. The inverted resistivity model falling below the depth of investigation (DOI), calculated from the sensitivity matrix of the final output model^48^, was removed. It gave a 3-D resistivity volume of the subsurface.

In addition, the smoothed inversion mode (SIM, 1-D resistivity-depth information) of the HTEM data was studied at the selective profiles where ground geophysical measurements (such as VES, ERT, and GTEM, etc.) were available to compare the layer parameters. Both VES and ERT data were collected with the aid of IRIS make Syscal R1 + instrument, France made, and the data were interpreted with help of IX1-D V.3 INTERPEX and Interrex and RES2DINV software. Whereas the GTEM data was collected with TEM 48 HPC, TerrTEM System, Netherlands make. The ground geophysical and HTEM results were correlated with borehole litho-logs, and finally, the hydrogeological inferences were drawn to deducing the multi-aquifer system up to the explored depth of 300 m and beyond. A typical hydrogeological section derived from the HTEM data^26,27,35^, had been deduced, combined of both ground and HTEM geophysical data as well as the hydrogeological inputs in the flown area. It has been observed that the result of the HTEM data clearly manifested the aquifer disposition concurring with the geophysical logs of boreholes drilled along with lithology, as shown in Fig. S2, and also satisfactorily matched with the VES, ERT, and GTEM results in the flown area^27^, as described in Figs. S3–S5 (in the Supplementary Information).

The GTEM, VES, and ERT results manifested the two-tiered aquifer system (such as shallow aquifer and first principal aquifer up to the explored depth of around 110 m), which are also agreeable with the results of the HTEM data in the flown area^27^. The flown area was triangular shape, an area of 150 km^2^ of the study area (~ 521 km^2^) covered by the HTEM survey due to non-availability of the clearances from the concerned Ministries. All groundwater geophysical data, including bore well informations, are used to interpret the layered earth. It helped to prepare 3D aquifer geometry for delineating three multi-aquifer system but the result has been extremely uncertain in the non-flown area due to dense data coverage. It has been observed a clear contrast among the clay layer (resistivity: up to 15 Ω-m) and sandy aquifers (resistivity: 15–120 Ω-m). The upper limit of the aquifer resistivity indicates the mixing of coarse sand/gravel in the study area. The first resistive layer at depths of around 25 m, bgl forming the first principal aquifer is clearly mapped. The second principal aquifer, which lies at an average 110 m depth in this area, is also demarcated. The geophysical properties such as thickness and resistivity of aquifer obtained from the HTEM survey (with the dense data, grid: 250 m × 250 m resolution) at the flown area was utilized for the projecting geophysical parameters such as the resistivity and thickness of aquifer to practical aquifer mapping in the non-flown area.

## Supplementary Information

Supplementary Information.

## Data Availability

The data sets both generated and analyzed during the current study are available from the corresponding authors on reasonable request.
