# Author Correction: Geostatistical spatial projection of geophysical parameters for practical aquifer mapping

**DOI:** 10.1038/s41598-022-23967-3

**Published:** 2022-11-16

**Authors:** Jagriti Dabas, Sarah Sarah, N. C. Mondal, Shakeel Ahmed

**Affiliations:** 1 TERI-SAS, Vasant Kunj, New Delhi, India; 2 Aquifer Mapping Group, CSIR-National Geophysical Research Institute, Hyderabad, 500007 India; 3 Department of Earth Sciences, University of Kashmir, Srinagar, 190006 India; 4 Earth Process Modeling Group, CSIR-National Geophysical Research Institute, Hyderabad, 500007 India; 5 Academy of Scientific and Innovative Research (AcSIR), Ghaziabad, 201002 India; 6 Maulana Azad National Urdu University, Hyderabad, 500032 India

Correction to: *Scientific Reports* 10.1038/s41598-022-08494-5, published online 06 April 2022

The Supplementary Information published with this Article contained errors in the ‘S3 Geomorphology’ section and in the legend of Figure S1. The original Supplementary Information is provided below.

Section ‘S3 Geomorphology’

“Although, the area represents a monotonously flat topography (Fig. S1), still a slight variation in topography indicates that the general slope is from southwest to northeast. The area is evacuated by the mighty river Ganges lying in the northern boundary of the project area. Rivers like Sone, Punpun and their tributaries are also major contributors to the cause of area drainage. The levee or upland is being constructed by river Ganges all along its southern bank. Majorly southern and western part of the study area is being traversed by at least 5 paleo-channels of Son River. Interestingly not a single stream flows northward or westward to join either River Ganges or Sone within the entire area of concern. The remains of Sone paleo-channels at patches presently serve as ‘pynes’31.”

now reads:

“Although, the area represents a monotonously flat topography (Fig. S1), still a slight variation in topography indicates that the general slope is from southwest to northeast. The area is evacuated by the mighty river Ganges lying in the northern boundary of the project area. Rivers like Son, Punpun and their tributaries are also major contributors to the cause of area drainage. The levee or upland is being constructed by river Ganges all along its southern bank. Majorly southern and western part of the study area is being traversed by at least 5 paleo-channels of Son River (CGWB, 2015). Interestingly not a single stream flows northward or westward to join either River Ganges or Son within the entire area of concern. The remains of Son paleo-channels at patches presently serve as ‘pynes’.”

The legend of Figure S1

“Geomorphological map of Patna, Bihar, Northern India (S.S.: drawn this figure considering LISS-III Image (November 2009), SRTM 90m data, ERDAS IMAGINE 9.3 module and digital image processing).”

now reads:

“Geomorphological map of the study area (CGWB, 2015, http://cgwb.gov.in/AQM/Pilot/Patna%20District,%20Bihar-Final.pdf).”

The Supplementary Information file has been corrected.

## Supplementary Information

Supplementary Information.

