# Supplementary material for: Author Correction: Geostatistical spatial projection of geophysical parameters for practical aquifer mapping
Source: Sci Rep. 2022 Nov 16;12:19688. doi: 10.1038/s41598-022-23967-3 (PMC9669009; doi:10.1038/s41598-022-23967-3)
Supplement: Supplementary file 1 — Supplementary Information. [file 41598_2022_23967_MOESM1_ESM.pdf]

Geostatistical spatial projection of geophysical parameters for practical aquifer mapping

Jagriti Dabas[a,b], Sarah Sarah[c, b, **], N.C. Mondal[d, e, b *] and Shakeel Ahmed[f,b]

[a]TERI-SAS, Vasant Kunj, New Delhi, India
[b]Aquifer Mapping Group, CSIR-National Geophysical Research Institute, Hyderabad-500007, India
[c]Department of Earth Sciences, University of Kashmir, Srinagar-190006, India
[d]Earth Process Modeling Group, CSIR-National Geophysical Research Institute, Hyderabad-500007, India
[e]Academy of Scientific & Innovative Research (AcSIR), Ghaziabad-201002, India

[f]Maulana Azad National Urdu University, Hyderabad-500032, India

*Corresponding author 1: N.C. Mondal (mondal@ngri.res.in, ncmngri@gmail.com)
**Corresponding author 2: Sarah Sarah (geosara1@gmail.com)

Contents of Supplementary Information

S1: Study area

S2: Geology and hydrodynamics of the aquifer system

S3: Geomorphology (as shown in Fig. S1)

S4: Showing (a) the HTEM flown area, and the comparative the results of HTEM with the borehole resistivity logs and lithology in the (b) N-S, and (c) DG sections, as shown in **Fig. S2**

S5: Showing the comparative results of HTEM and GTEM data, as shown **Fig. S3**

S6: Showing the comparative results of HTEM and VES data, as shown **Fig. S4**

S7: Showing the comparative results of HTEM and ERT data, as shown **Fig. S5**

S1 Study area

The research area is situated in the western and south-western parts of Patna city, Northern India, and is located on the southern bank of river holy Ganga. The study area spreads over 521 km^2, in the Middle Ganges Plain (**Fig. 1**). The area lies between 25°25′12″ N and 25°40′48″ N to 84°49′12″ E and 85°13′12″ E. It is bounded by three rivers such as river Ganga in the northern part, Son river in the western part, and Punpun river in the southern part of the study area.

S2 Geology and hydrodynamics of the aquifer system

The area constitutes of thick alluvial deposits forming a part of Gangetic plain resting over the pre-Tertiary formations, which comprise sediments like sand, gravel, and clay. It comprises a multi-aquifer system. Shallow surface paleo-channels represent a typical sedimentary river basin originated by the network of Son and Ganges rivers. The aquifer lying immediately under this clay capping surface of varying thickness is known as 'first principal aquifer' that extends up to depths of around 100 m. In the northern part along the course of River Ganges, the first principal aquifer is arsenic contaminated at several places. The underlying layer of second principal aquifer is arsenic free. Near surface aquifers are formed by paleo-channels which are also the localized reservoir of fresh groundwater (CGWB, 2015).

As discussed above, the area being an alluvial formation, there are alternate layers of sands of different sizes separated by clay. From the deep drilling as well as few deep soundings, it is established that three aquifer layers separated by the clay of varying thickness form the entire aquifer system. The third deep aquifer has not been extensively explored. Also, the area being very close to Son-Ganges river system, enormous surface water is available. Hence the first principal aquifer that is comparatively shallow and unconfined in nature has been extensively exploited. The confining or the separation clay layer vary a lot in thickness and allows leakage

of water between the two aquifers, and this leakage depends on the thickness and difference of hydraulic heads of the two aquifer layers. Before the extensive exploitations, it was found that groundwater leakage has been from the second aquifer to the first aquifer, but after the uncontrolled and unmanaged exploitation from the second aquifer, the hydrodynamics has been changing, and reversal of leakage might take place (Mondal et. al., 2019). Also, recent studies explain that due to the presence of arsenic in newer sediments, the first principal aquifer is extensively contaminated with arsenic, and the second principal aquifer that is confined with clay layer is free from arsenic as the arsenic is not found in the older sediments, the basic formation of the second principal aquifer. Thus, there is heavy pumping from the second principal aquifer that can alter the leakage between the aquifers.

S3 Geomorphology

Although, the area represents a monotonously flat topography (**Fig. S1**), still a slight variation in topography indicates that the general slope is from southwest to northeast. The area is evacuated by the mighty river Ganges lying in the northern boundary of the project area. Rivers like Sone, Punpun and their tributaries are also major contributors to the cause of area drainage. The levee or upland is being constructed by river Ganges all along its southern bank. Majorly southern and western part of the study area is being traversed by at least 5 paleo-channels of Son River. Interestingly not a single stream flows northward or westward to join either River

Ganges or Sone within the entire area of concern. The remains of Sone paleo-channels at patches presently serve as ‘pynes’31.

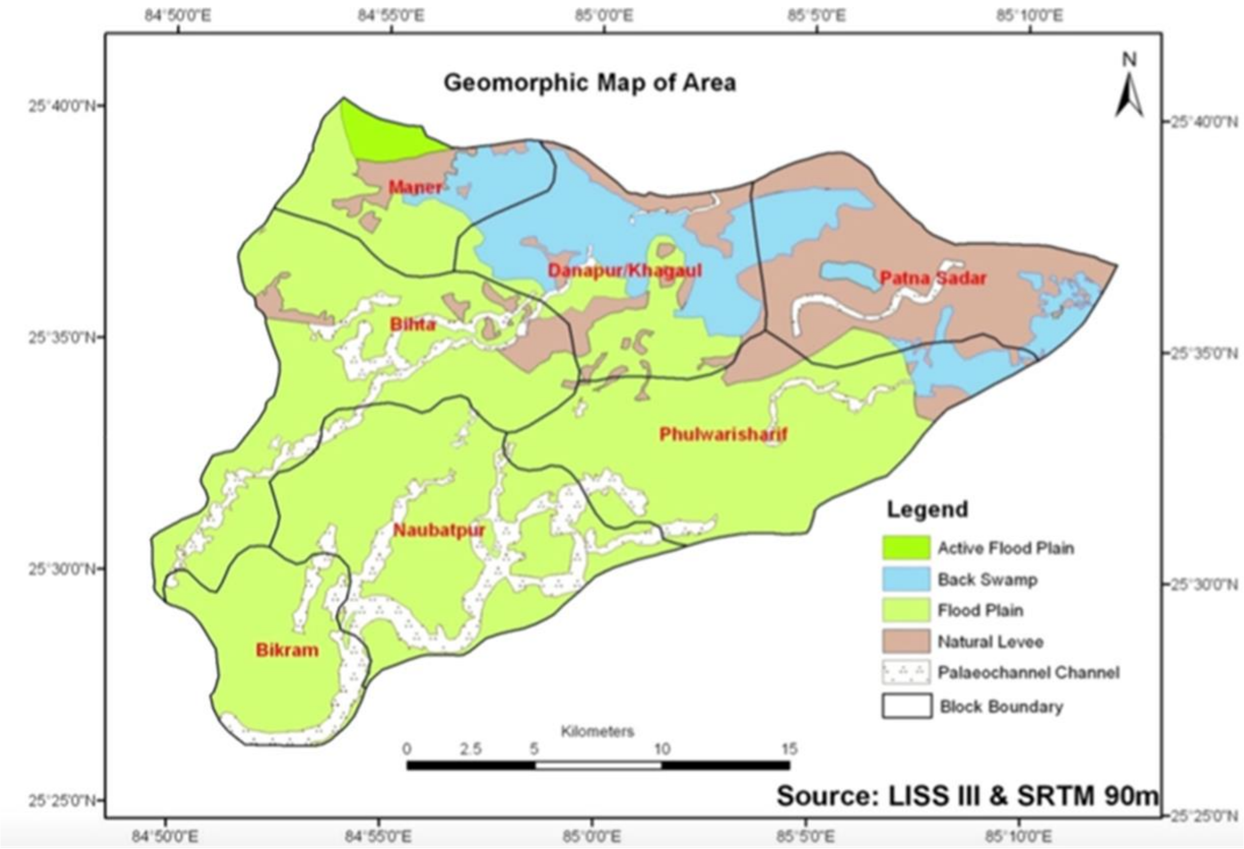

Figure S1. Geomorphological map of Patna, Bihar, Northern India (S.S.: drawn this figure considering LISS-III Image (November 2009), SRTM 90m data, ERDAS IMAGINE 9.3 module and digital image processing)

S4: The HTEM flown area, and the comparative the results of HTEM with the borehole resistivity logs and lithology in the flown area, as shown in **Fig. S2**

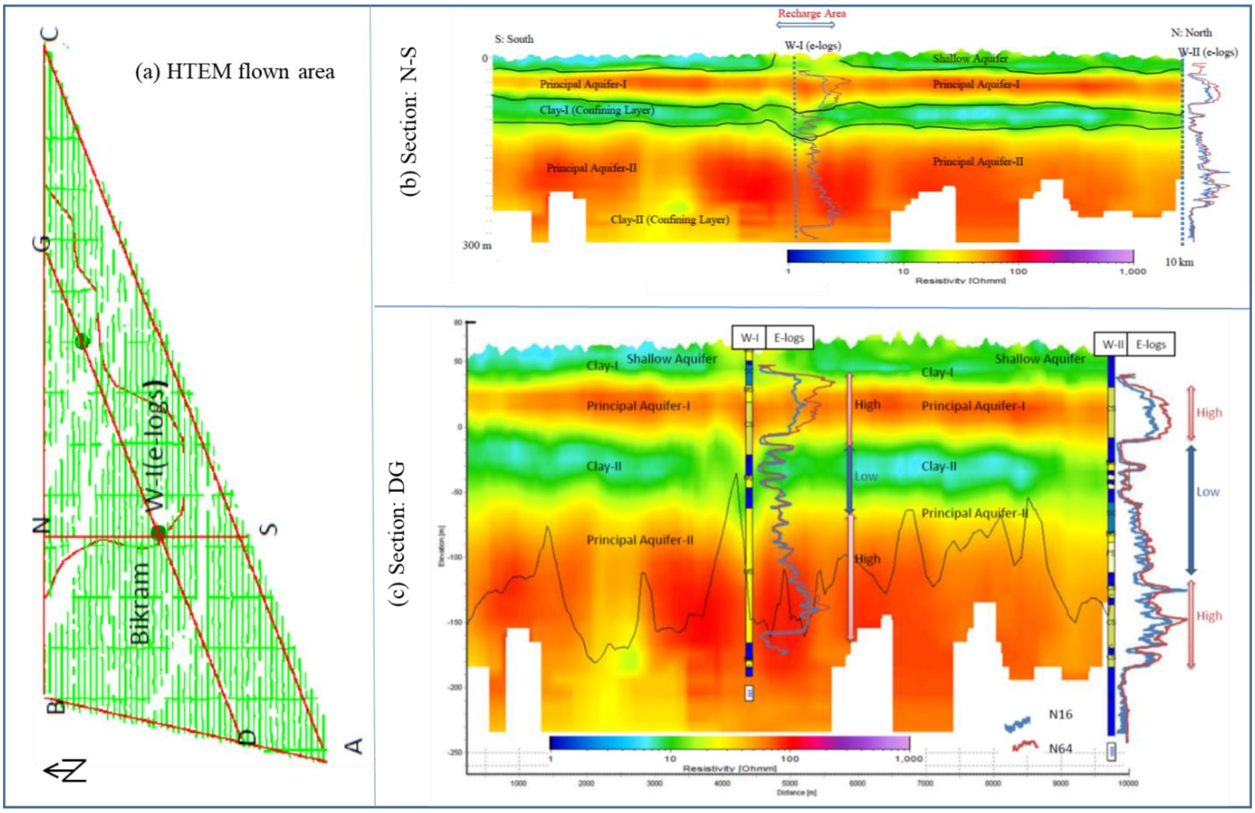

Figure S2. Showing (a) the HTEM flown area, and comparison of the results of HTEM with the resistivity log of boreholes with 2-D resistivity distribution in the (b) N-S section, and (c) DG section (N.C.M.: sketched this figure of HTEM data with the aid of Aarhus Workbench**,** https://www.aarhusgeosoftware.dk)

The above figure has been deduced combined of both ground and HTEM geophysical data as well as the hydrogeological inputs in the flown area. It has been observed that the result of the HTEM data clearly manifested the aquifer disposition concurring with the geophysical logs of boreholes drilled in the flown area (Mondal et al., 2015b; Ahmed et al., 2015).

S5: Comparative results of HTEM and GTEM data in the flown area

The GTEM data at the site of TEM 17 (village Bikram) indicates clay (resistivity:17 Ω-m) up to the depth of 28 m bgl (**Fig. S3**). The near-surface aquifer is obtained within 9 m depth. The bottom of first principal aquifer (resistivity:58Ω-m) is at 77 m bgl. Underlying this the clay layer (resistivity: 12 Ω-m) is encountered. The bottom of the first aquifer is not resolved by the GTEM data alone due to low power transmitter of the equipment (TEM Fast 48 PC) used. The resistivity around 17 Ω-m for the clay is also obtained from HTEM SIM (GDI: 230m), but the resistivity of the underlying first principal aquifer is less compared to the GTEM data. The depth to the bottom of first principal aquifer obtained from the HTEM and the GTEM data are in agreement (Mondal et al., 2015b).

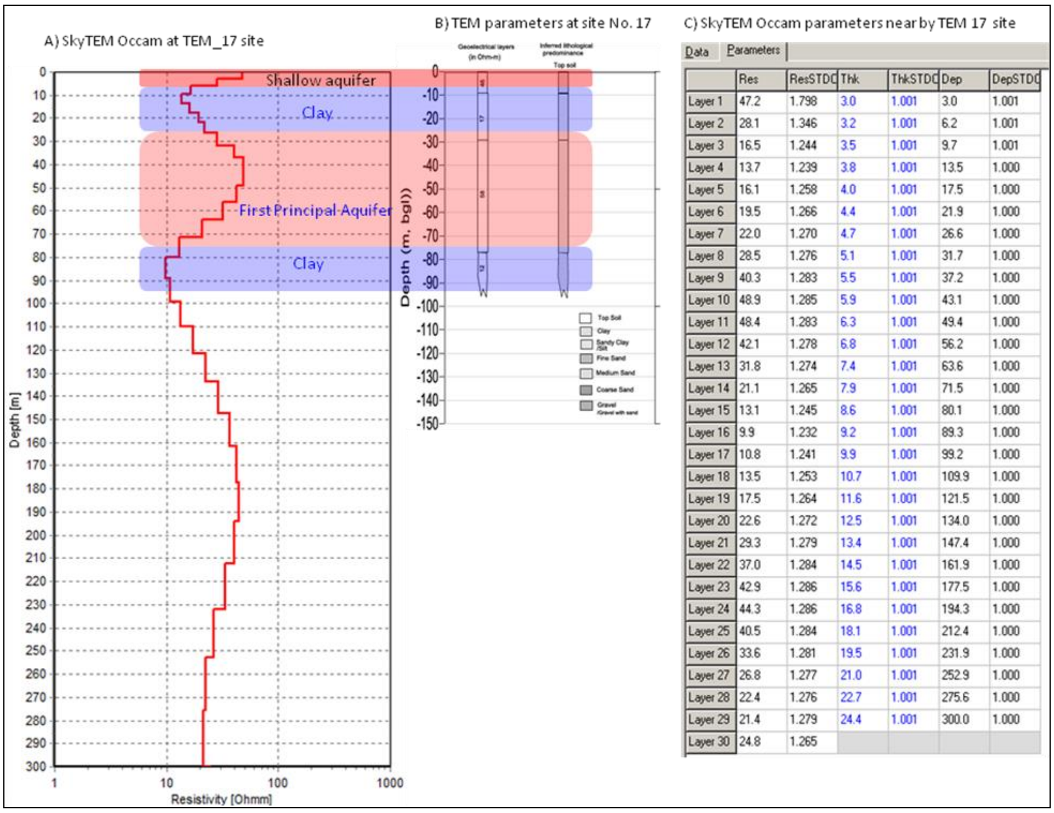

	Res	ResSTDC	Thk	ThkSTDC	Dep	DepSTDC
Layer 1	47.2	1.798	3.0	1.001	3.0	1.001
Layer 2	28.1	1.346	3.2	1.001	6.2	1.001
Layer 3	16.5	1.244	3.5	1.001	9.7	1.001
Layer 4	13.7	1.239	3.8	1.001	13.5	1.000
Layer 5	16.1	1.258	4.0	1.001	17.5	1.000
Layer 6	19.5	1.266	4.4	1.001	21.9	1.000
Layer 7	22.0	1.270	4.7	1.001	26.6	1.000
Layer 8	28.5	1.276	5.1	1.001	31.7	1.000
Layer 9	40.3	1.283	5.5	1.001	37.2	1.000
Layer 10	48.9	1.285	5.9	1.001	43.1	1.000
Layer 11	48.4	1.283	6.3	1.001	49.4	1.000
Layer 12	42.1	1.278	6.8	1.001	56.2	1.000
Layer 13	31.8	1.274	7.4	1.001	63.6	1.000
Layer 14	21.1	1.265	7.9	1.001	71.5	1.000
Layer 15	13.1	1.245	8.6	1.001	80.1	1.000
Layer 16	9.9	1.232	9.2	1.001	89.3	1.000
Layer 17	10.8	1.241	9.9	1.001	99.2	1.000
Layer 18	13.5	1.253	10.7	1.001	109.9	1.000
Layer 19	17.5	1.264	11.6	1.001	121.5	1.000
Layer 20	22.6	1.272	12.5	1.001	134.0	1.000
Layer 21	29.3	1.279	13.4	1.001	147.4	1.000
Layer 22	37.0	1.284	14.5	1.001	161.9	1.000
Layer 23	42.9	1.286	15.6	1.001	177.5	1.000
Layer 24	44.3	1.286	16.8	1.001	194.3	1.000
Layer 25	40.5	1.284	18.1	1.001	212.4	1.000
Layer 26	33.6	1.281	19.5	1.001	231.9	1.000
Layer 27	26.8	1.277	21.0	1.001	252.9	1.000
Layer 28	22.4	1.276	22.7	1.001	275.6	1.000
Layer 29	21.4	1.279	24.4	1.001	300.0	1.000
Layer 30	24.8	1.265				

Figure S3: The SIM results of HTEM data at the site of GTEM 17; A) SIM of HTEM sounding, B) Layer parameters of the GTEM and inferred litho-units, and C) interpreted HTEM SIM parameters at TEM 17 site (N.C.M.: sketched this figure of HTEM data with the aid of Aarhus Workbench**,** https://www.aarhusgeosoftware.dk)

S6: Comparative results of HTEM and VES data in the flown area

The VES-3 observed at Bikram is located towards east of Bikram bore well (W-I). The interpretation of the VES curve yielded 9 geoelectrical layers (**Fig.S4**) and the depth investigated is beyond 526 m. The VES reveals a clay with 8 Ω-m resistivity in the depth range of 14 to 25 m bgl. The overlying layer has a higher resistivity around 27Ω-m. At this site the first principal aquifer is associated with 58 Ω-m resistivity. Its thickness is around 54 m which matches satisfactorily with the HTEM resistivity section (**Fig.S4**). This layer is underlain by a conductive layer with 28Ω-m and may form the confining layer. Its thickness is ~54 m. This is further underlain by a layer with 51 Ω-m resistivity at a depth of 133m. It is the second principal aquifer of thickness around 96m (Mondal et al., 2015b).

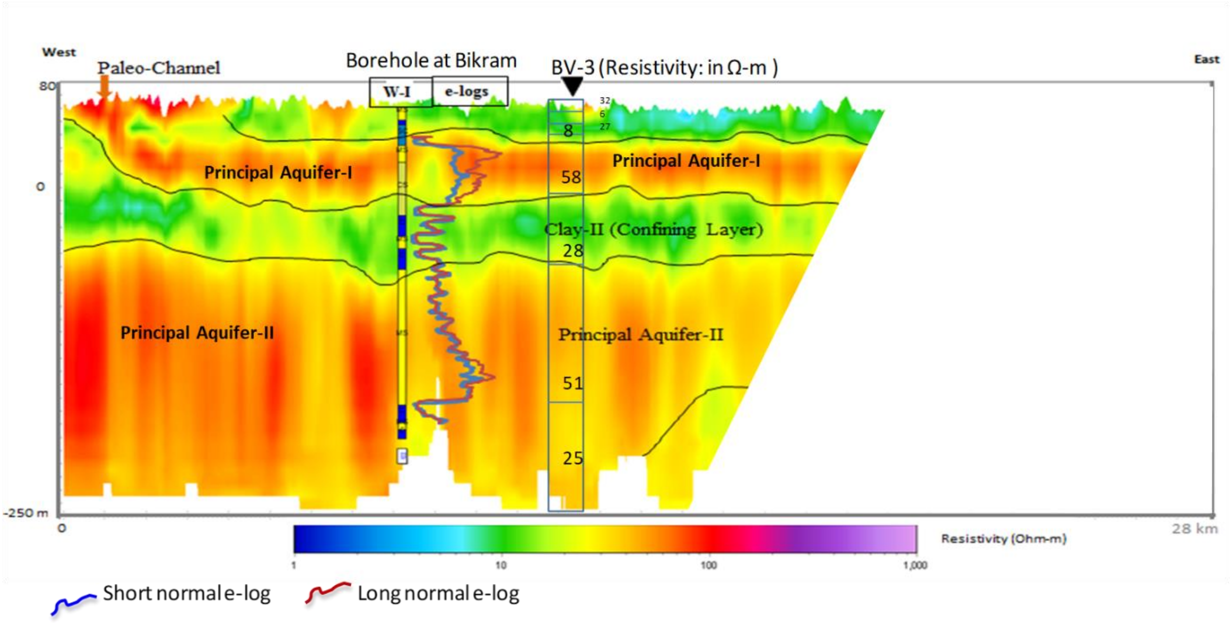

Figure S4: The HTEM results compared with the results of VES (BV-3) in the flown area of Northern India (N.C.M.: sketched this figure of HTEM data with the aid of Aarhus Workbench**,** https://www.aarhusgeosoftware.dk)

S7: Comparative results of HTEM and ERT data in the flown area

The ERT (**Fig. S5),** carried out in the HTEM flown area, revealed a clay layer up to an average depth of 26 m bgl and thereafter a sand layer - the first principal aquifer continuing to the explored depth of 60 m along this profile. It was confirmed through the HTEM data shown in **Fig. S5B**. The HTEM sounding Occam inversion result at the centre of the ERT profile indicates first principal aquifer occurring at a depth of 26 m bgl and continuing up to 60m bgl. Its resistivity varies from 25 to 68 Ω-m. It is also observed that the resistivity of top clay layer varies from 8.6 to 15.1Ω-m with an average resistivity of 12 Ω-m in the HTEM Occam inversion (**Fig. S5C**) which is more or less same (as in the ERT results). The clay layer at the bottom of the first principal aquifer acting as a barrier between the two principal aquifers was not resolved through ERT data due to the limitation of instrument and spread constraint (Mondal et al., 2015b).

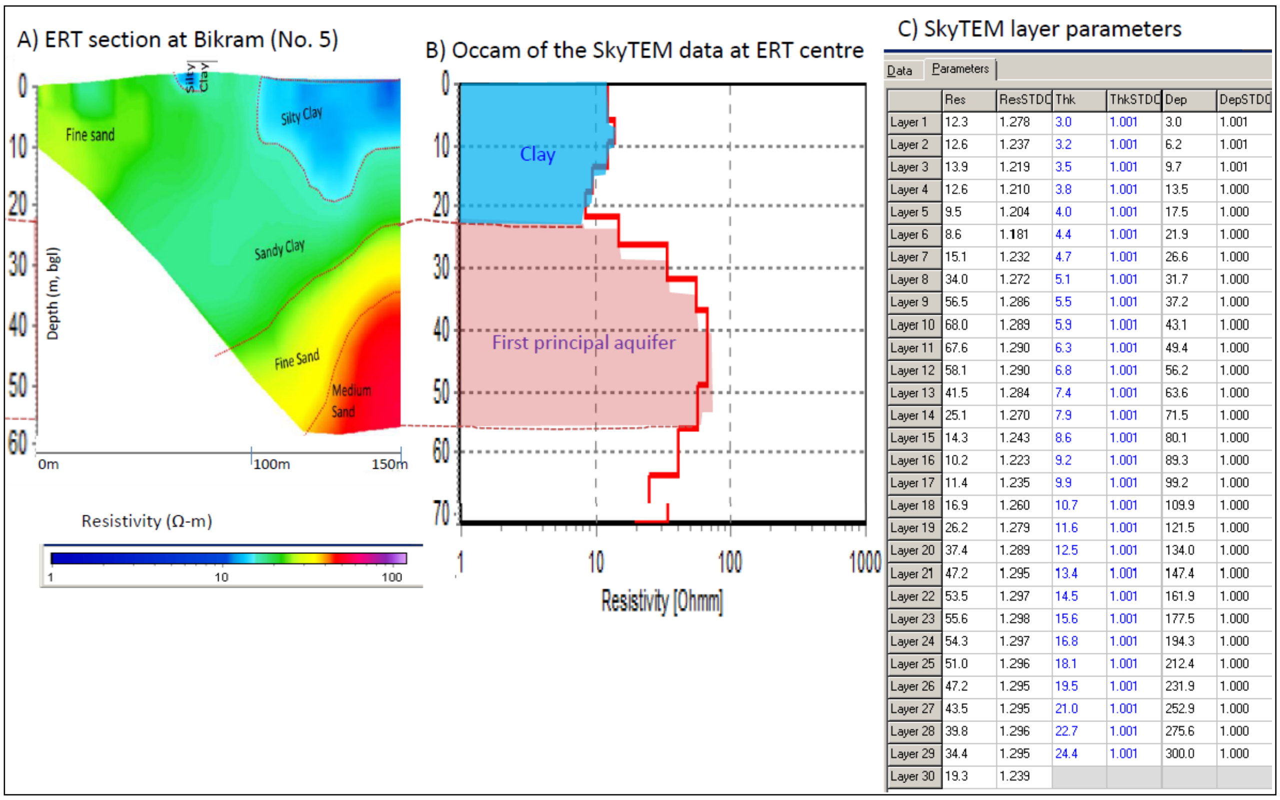

	Res	ResSTDC	Thk	ThkSTDC	Dep	DepSTDC
Layer 1	12.3	1.278	3.0	1.001	3.0	1.001
Layer 2	12.6	1.237	3.2	1.001	6.2	1.001
Layer 3	13.9	1.219	3.5	1.001	9.7	1.001
Layer 4	12.6	1.210	3.8	1.001	13.5	1.000
Layer 5	9.5	1.204	4.0	1.001	17.5	1.000
Layer 6	8.6	1.181	4.4	1.001	21.9	1.000
Layer 7	15.1	1.232	4.7	1.001	26.6	1.000
Layer 8	34.0	1.272	5.1	1.001	31.7	1.000
Layer 9	56.5	1.286	5.5	1.001	37.2	1.000
Layer 10	68.0	1.289	5.9	1.001	43.1	1.000
Layer 11	67.6	1.290	6.3	1.001	49.4	1.000
Layer 12	58.1	1.290	6.8	1.001	56.2	1.000
Layer 13	41.5	1.284	7.4	1.001	63.6	1.000
Layer 14	25.1	1.270	7.9	1.001	71.5	1.000
Layer 15	14.3	1.243	8.6	1.001	80.1	1.000
Layer 16	10.2	1.223	9.2	1.001	89.3	1.000
Layer 17	11.4	1.235	9.9	1.001	99.2	1.000
Layer 18	16.9	1.260	10.7	1.001	109.9	1.000
Layer 19	26.2	1.279	11.6	1.001	121.5	1.000
Layer 20	37.4	1.289	12.5	1.001	134.0	1.000
Layer 21	47.2	1.295	13.4	1.001	147.4	1.000
Layer 22	53.5	1.297	14.5	1.001	161.9	1.000
Layer 23	55.6	1.298	15.6	1.001	177.5	1.000
Layer 24	54.3	1.297	16.8	1.001	194.3	1.000
Layer 25	51.0	1.296	18.1	1.001	212.4	1.000
Layer 26	47.2	1.295	19.5	1.001	231.9	1.000
Layer 27	43.5	1.295	21.0	1.001	252.9	1.000
Layer 28	39.8	1.296	22.7	1.001	275.6	1.000
Layer 29	34.4	1.295	24.4	1.001	300.0	1.000
Layer 30	19.3	1.239				

Figure S5: Showing the comparison of ERT and HTEM results; A) ERT section carried out at Bikram (BE-5), B) the SIM of the HTEM sounding data at the center of the ERT and

C) interpreted SIM parameters (N.C.M.: sketched this figure of HTEM data with the aid of Aarhus Workbench**,** https://www.aarhusgeosoftware.dk)

References:

Ahmed, S., Chandra, S., Verma, S.K., Muralidharan, Mondal, N.C., Somvanshi, V.K., Venkatarayudu, M., Ramadevi, A., Tiwari, R.K., Veerababu, N., Maurya, P.K. and Jaiswal, D.K. (2015). *Final report on pilot aquifer mapping project (AQUIM): AQBHR, Patna District, Bihar*. Restricted Technical Report No.: NGRI-2015-GW-892, p.133 (2015).

Central Ground Water Board (CGWB). Final draft report on Pilot Project on Aquifer Mapping in Maner-Khagaul Area, Patna District, Bihar, (Watershed -GNDK013). (2015).http://www.cgwb.gov.in/AQM/Pilot/Patna%20District,%20Bihar-Final.pdf

Mondal, N.C. et al. *Hydrogeological Inferences using Heliborne Transient Electromagnetic and Ground Geophysical data in AQBHR Area, Patna District, Bihar, India*. Restricted Technical Report No.: NGRI-2015-GW-880, April 2015, p.102 (2015b).

Mondal, N.C., Surinaidu, L., Ahmed, S., Tiwari, V.M. A multi-layered groundwater model for leakage assessment of arsenic contamination threat in a part of Ganga basin, In: Waterfuture Conference "*Towards a Sustainable Water Future*" held on September 24-27, 2019 at Bengaluru, India (2019).
